# The protective model of myofascial trigger points: a testable systems-level framework based on strain-repair mismatch

**DOI:** 10.3389/fphys.2026.1818589

**Published:** 2026-05-07

**Authors:** Michael Strickland, Micheal J. Luera, Mandy E. Parra, Chris Riggs

**Affiliations:** 1 College of Health and Clinical Professions, Division of Health Sciences, Tarleton State University, Stephenville, TX, United States; 2 College of Health and Clinical Professions, Division of Health Sciences, College of Science and Mathematics, Department of Neuroscience, Physiology Research of Integrative Muscle and Electrophysiology Laboratory, Tarleton State University, Stephenville, TX, United States; 3 Division of Health Sciences, Physiology Research of Integrative Muscle and Electrophysiology Laboratory, Tarleton State University, Stephenville, TX, United States

**Keywords:** adaptive sentinel response, muscle physiology, musculoskeletal pain, myofascial trigger points, neuromuscular physiology, protective model, strain–repair mismatch, tissue repair

## Abstract

Myofascial trigger points (MTPs) are frequently identified in clinical practice, yet their underlying physiology remains conceptually fragmented across biochemical, mechanical, and neurophysiological domains. Commonly reported features, including spontaneous electrical activity, increased stiffness, nociceptive sensitization, hypoxia, and elevated inflammatory mediators, are well documented but are not consistently interpreted within established frameworks of tissue stress and repair biology. This paper proposes a systems-level integrative framework, the Protective Model of Myofascial Trigger Points, which conceptualizes MTPs as localized tissue responses emerging when cumulative mechanical strain exceeds local repair capacity. Within this model, increased stiffness, sustained motor endplate activity, nociceptive sensitization, and localized neuroimmune signaling are interpreted as coordinated responses to strain–repair imbalance rather than independent pathological abnormalities. Variability in clinical presentation, including atent and active classifications, is proposed to reflect differences in the magnitude and duration of mechanical overload rather than biologically distinct entities, with persistent MTPs representing indicators of unresolved load–recovery mismatch. Eight falsifiable predictions are outlined, addressing load modification, recurrence patterns, elastographic stiffness, electromyographic behavior, biochemical signatures, and temporal resolution dynamics, providing explicit criteria for empirical testing. By situating MTP-associated phenomena within established principles of tissue injury, repair, and neuromuscular stress physiology, the Protective Model offers a coherent framework to guide systematic experimental investigation of MTP formation, persistence, and resolution.

## 1 Introduction

Myofascial trigger points (MTPs) have long been a subject of clinical and theoretical debate, characterized by persistent conceptual ambiguity and a lack of consensus regarding their definition, diagnosis, and underlying physiology (Quintner et al., 2015; Barbero et al., 2019). Even their symptomatology is described variably across the literature. Commonly reported features include localized hyperirritability within skeletal muscle, the presence of a palpable taut band, tenderness to palpation, local twitch response, and referred pain patterns (Shah et al., 2015; Gerwin, 2023). However, some MTPs present without spontaneous tenderness and are instead classified as latent trigger points, further complicating diagnostic categorization and clinical interpretation (Bron and Dommerholt, 2012; Ge et al., 2012; Hadizadeh et al., 2025).

A growing body of research has identified biochemical and electrophysiological correlates within MTP regions, including elevated concentrations of substance P, bradykinin, calcitonin gene-related peptide, pro-inflammatory cytokines, and spontaneous endplate activity (Dommerholt et al., 2019; Ge et al., 2009; Jafri, 2014; Shah et al., 2015; Lv and Yin, 2024). These findings are frequently accompanied by localized ischemia, hypoxia, increased tissue stiffness, and altered motor control patterns (Ballyns et al., 2012; Jin et al., 2020; Itoh et al., 2004; Silva et al., 2025; Shah et al., 2015). Collectively, these observations indicate that MTP regions demonstrate measurable physiological activity rather than representing purely inert structural irregularities.

Despite the relative consistency of these physiological findings, their interpretation varies substantially across the literature. Existing frameworks alternately conceptualize MTPs as manifestations of peripheral nociceptive dysfunction, sensitization processes, neuromuscular dysregulation, or nonspecific diagnostic constructs (Fernández-de-las-Peñas et al., 2007; Stieven et al., 2021; Barbero et al., 2019; Quintner et al., 2015). This conceptual divergence has contributed to ongoing controversy regarding MTP diagnosis, explanatory mechanisms, and optimal treatment approaches.

To address this fragmentation, the present manuscript reconceptualizes MTPs as biologically mediated protective responses to sustained tissue stress. Within the Protective Model, MTPs are understood not as random pathological errors but as patterned physiological responses that emerge when cumulative mechanical strain exceeds local tissue repair capacity.

In this manuscript, adaptive sentinel response will be used frequently and refers to a coordinated, non-conscious physiological pattern that emerges under conditions of strain–repair mismatch, characterized by load-modulating neuromuscular, sensory, biochemical, and structural changes rather than intentional or goal-directed tissue behavior.

Within this framework, MTPs represent integrated musculoskeletal, neurological, and immune responses to unresolved tissue stress. Their clinical presentation therefore reflects the magnitude and persistence of overload rather than strictly discrete latent and active categories, with resolution expected following correction of the underlying strain–repair imbalance.

The Protective Model is a biologically grounded, process-based explanatory framework that integrates neuromuscular, biochemical, and sensory findings reported in the MTP literature. It does not imply a singular, experimentally verified causal pathway, nor is it limited to mechanical tissue properties alone. Rather, the model provides a systems-level framework derived from converging empirical observations, in which MTP formation becomes likely under identifiable conditions of strain–repair mismatch. In this sense, the Protective Model treats MTP formation as a stochastic physiological process: strain–repair mismatch increases the probability of adaptive sentinel response emergence, but its expression is probabilistic rather than uniform. Accordingly, chronically persistent MTPs are interpreted within this model as indicators of unresolved strain–repair mismatch, in which tissue stress continues to exceed local regenerative capacity.

## 2 Historical and clinical context

The concept of MTPs originates from the mid-20th-century work of Travell and Simons, who described them as discrete palpable nodules located within taut bands of skeletal muscle fibers and frequently associated with referred pain patterns and localized tenderness. These early clinical observations were progressively formalized into diagnostic criteria that continue to be widely cited in contemporary literature despite ongoing lack of consensus regarding the underlying biological processes responsible for these findings (Gerwin, 2023; Shah et al., 2015).

The diagnostic framework gained clinical traction due to its apparent simplicity and practical applicability. However, subsequent research raised concerns regarding inter-examiner reliability, symptom specificity, and the limited capacity of conventional imaging modalities to consistently visualize MTP structures (Quintner et al., 2015; Sikdar et al., 2009; Wilke et al., 2018). Concurrently, methodological critiques have highlighted the potential for circular attribution, wherein the presence of pain supports classification of active trigger points, while the absence of spontaneous pain supports classification of latent trigger points, thereby reducing the falsifiability of the construct.

Clinically, MTPs are reported across a wide range of acute and chronic musculoskeletal conditions, including postural strain syndromes, overuse injuries, fibromyalgia, temporomandibular disorders, cervicogenic headache, and low back pain (Dommerholt et al., 2019; Stieven et al., 2021). Nevertheless, their precise role within these conditions remains unresolved, as it is unclear whether MTPs function as primary drivers of pain, secondary responses to underlying tissue stress, or epiphenomena associated with broader neuromusculoskeletal dysfunction. The distinction between active and latent MTPs further complicates interpretation, particularly as latent points may not produce spontaneous pain yet are still associated with altered muscle function and increased fatigability (Bron and Dommerholt, 2012; Ge et al., 2012; Hadizadeh et al., 2025).

A substantial body of literature associates MTPs with reduced range of motion, motor dysfunction, localized and referred pain, and facilitation of central sensitization (Bron and Dommerholt, 2012; Dommerholt et al., 2019; Fernández-de-las-Peñas et al., 2007; Ge et al., 2009; Shah et al., 2015; Silva et al., 2025; Stieven et al., 2021). Accordingly, several authors advocate for routine assessment of MTP presence in patients with nonspecific musculoskeletal pain due to their high prevalence and documented responsiveness to targeted interventions (Jafri, 2014; Gerwin, 2023).

Despite decades of clinical characterization and increasing identification of associated biochemical, electrophysiological, and mechanical features, the field still lacks a unifying biologically grounded explanatory framework that integrates these findings within established principles of tissue stress and repair physiology. This persistent conceptual fragmentation, occurring alongside converging physiological observations, underscores the need for a systems-level theoretical integration. Reframing MTP-related phenomena within the context of stress–repair dynamics provides a biologically coherent foundation for interpreting their formation, persistence, and resolution.

## 3 Physiological features commonly attributed to trigger points

### 3.1 Neuromuscular activity

Multiple studies have reported spontaneous electrical activity in regions corresponding to MTPs (Butts et al., 2016; Ge et al., 2009; Holanda et al., 2015; Jafri, 2014; Shah et al., 2015). Intramuscular electromyography demonstrates persistent endplate noise and abnormal miniature endplate potentials, interpreted as excessive acetylcholine release or impaired neuromuscular junction regulation. This dysregulation is hypothesized to contribute to sustained local contractile activity and taut band formation (Ge et al., 2009; Holanda et al., 2015; Jafri, 2014; Shah et al., 2015).

Notably, while spontaneous electrical activity increases within MTP regions, voluntary motor unit recruitment within the affected zone demonstrates reduced amplitude relative to adjacent healthy tissue (Gerwin, 2023). Prevailing interpretations suggest that persistent depolarization or dysfunctional repolarization at the motor endplate may sustain localized contracture in a subset of fibers.

### 3.2 Biochemical and microenvironmental features

Substance P, calcitonin gene-related peptide, bradykinin, tumor necrosis factor alpha, and interleukins such as IL-1β and IL-6 have been identified in elevated concentrations within active and latent MTP regions (Butts et al., 2016; Hadizadeh et al., 2025; Holanda et al., 2015; Shah et al., 2015; Sadria et al., 2016). These mediators are associated with nociceptor sensitization, vasomotor regulation, immune signaling, and inflammatory modulation, supporting the hypothesis that MTPs represent localized neuroimmune microenvironments.

Imaging and tissue sampling studies further demonstrate hypoxic and ischemic characteristics within MTP regions, including reduced capillary perfusion and diminished oxygenation (Ballyns et al., 2012; Jin et al., 2020). Such metabolic stress may promote both sustained contractile activity and accumulation of inflammatory mediators, suggesting a feedback interaction between energetic deficit and nociceptive sensitization.

### 3.3 Sensory and central contributions

Peripheral sensitization is consistently observed within MTP regions, reflected in reduced nociceptive thresholds and enhanced local pain responses (Fernández-de-las-Peñas et al., 2007; Shah et al., 2015). Repeated nociceptive input from these regions may facilitate central sensitization through spinal and supraspinal amplification pathways, contributing to referred pain patterns and expanded zones of hyperalgesia (Dommerholt et al., 2019; Suresh et al., 2021). These combined peripheral and central mechanisms help explain the clinical observation that pain intensity may exceed the apparent extent of structural involvement.

### 3.4 Mechanical and structural features

MTPs are classically associated with palpable taut bands, often attributed to sustained sarcomere shortening or persistent motor endplate hyperactivity with impaired calcium reuptake (Ballyns et al., 2012; Gerwin, 2023). Elastographic and ultrasonographic studies demonstrate reduced tissue compliance and increased localized stiffness compared to surrounding uninvolved muscle (Silva et al., 2025; Szajkowski et al., 2025; Wilke et al., 2018).

Histological and biochemical analyses have identified acetylcholinesterase accumulation near motor endplates, elevated Substance P expression, mitochondrial irregularities, and markers of oxidative stress (Jafri, 2014; Sadria et al., 2016; Shah et al., 2015). Microscopic examination of affected fibers demonstrates sarcomere disorganization, micro-tearing, and localized inflammatory cell presence, consistent with increased metabolic demand and impaired recovery.

### 3.5 Summary

Despite extensive characterization of neuromuscular, biochemical, metabolic, and structural features associated with MTPs, interpretations of these findings remain fragmented. Spontaneous electrical activity, inflammatory mediators, tissue contracture, and hypoxia are frequently described as discrete pathological abnormalities rather than components of a unified physiological process. Although several models have proposed cyclical dysfunction or localized energy crisis mechanisms, these explanations often emphasize perpetuating loops without clearly situating the phenomena within broader principles of tissue stress and repair physiology (Gerwin, 2023).

To date, no widely accepted framework has systematically integrated the electrical, biochemical, mechanical, and nociceptive dimensions of MTPs into a single, testable explanatory model. This absence of integration has left the field with extensive descriptive data but limited consensus regarding the functional and biological significance of MTP formation and persistence.

## 4 Review of prevailing models

Prevailing models of MTPs have developed across multiple domains, including neuromuscular physiology, pain science, and muscle pathology. Rather than a single unified explanatory framework, the literature conceptualizes MTPs through several overlapping explanatory lenses, most commonly neuromuscular dysfunction, metabolic stress (energy crisis), peripheral and central sensitization, and localized biochemical alterations. These models do not necessarily contradict one another, but instead emphasize different primary physiological processes, ranging from motor endplate activity and nociceptive processing to inflammatory signaling and tissue remodeling. Collectively, they provide partial explanations for the clinical presentation, persistence, and symptom variability associated with MTPs.

### 4.1 Neuromuscular dysfunction and motor endplate models

Within the neuromuscular dysfunction framework, SEA and sustained contracture within taut bands are commonly interpreted as indicators of abnormal motor endplate activity and localized sarcomere shortening. Electrophysiological studies have consistently demonstrated increased endplate noise and spontaneous electrical discharges in regions identified as MTPs, suggesting altered neuromuscular regulation in affected muscle tissue (Jafri, 2014; Shah et al., 2015). These findings have been associated with persistent localized contraction, increased mechanical sensitivity, and motor unit hyperexcitability. Additionally, muscle guarding and spasmodic responses observed following acute muscle injury have been proposed as relevant physiological parallels, indicating that sustained contraction may occur in response to tissue insult or mechanical stress (Chapman et al, 2016; Sant’Anna et al., 2022; Ge et al., 2025).

### 4.2 Biochemical and neuroimmune models of MTPs

Biochemical investigations of MTP regions consistently report elevated concentrations of inflammatory and nociceptive mediators, including Substance P, bradykinin, CGRP, IL-6, TNF-α, and IL-1β, supporting the presence of an active neuroimmune microenvironment (Shah et al., 2015; Sadria et al., 2016; Hadizadeh et al., 2025; Su and Xiang, 2025). These mediators are known to facilitate nociceptor sensitization, vasodilation, immune cell recruitment, and local tissue signaling, processes commonly associated with muscle repair and inflammatory regulation (Forcina et al., 2020; Tidball, 2017). The detection of these substances in both active and latent MTPs has led to the interpretation that trigger point regions represent sites of ongoing biochemical activity rather than inert tissue abnormalities. Refer to **Table 1** for a visualization of the biochemical mediators most consistently reported in MTP literature.

**Table 1:** Biochemical markers in MTPs.

Biochemical marker	Primary roles	Relevance in MTP
Substance P	Pain facilitation, vasodilation, immune cell recruitment	Elevated in active MTP sites; contributes to local hyperalgesia
Bradykinin	Nociceptor sensitization, vasodilation, plasma extravasation	Frequently elevated in MTP tissue; promotes sensitization and inflammation
CGRP	Neurogenic inflammation, vasodilation, nociceptor modulation	Found in both latent and active MTPs; associated with peripheral sensitization
IL-6	Pro-inflammatory cytokine, supports immune signaling and tissue response	Elevated in trigger point microenvironments; supports immune crosstalk
TNF-α	Initiates inflammation, recruits immune cells, primes tissue repair	Detected in MTP biopsies; acts as an early signal in muscle healing
IL-1β	Coordinates innate immune response, promotes fibroblast and ECM activity	Amplifies inflammatory cascade and supports tissue remodeling in MTP zones

Hypoxia and localized ischemia have also been frequently described within MTP regions, particularly in association with sustained contraction and reduced perfusion (Ge et al., 2025; Jin et al., 2020; Su and Xiang, 2025). Within the energy crisis or integrated hypothesis, these metabolic conditions are proposed to contribute to ATP depletion, impaired calcium reuptake, and persistent contracture, reinforcing localized tissue stress and nociceptive signaling.

### 4.3 Peripheral and central sensitization models

Sensitization-based models, including both peripheral and central mechanisms, conceptualize MTPs as significant nociceptive sources capable of driving altered sensory processing and pain amplification. Elevated levels of inflammatory mediators and sustained nociceptor activation may lower pain thresholds and contribute to hyperalgesia and referred pain patterns (Dommerholt et al., 2019; Shah et al., 2015). Central sensitization models further propose that ongoing nociceptive input from MTPs may facilitate changes in spinal and supraspinal processing, thereby amplifying pain perception and contributing to chronic musculoskeletal pain conditions. From a functional perspective, pain associated with MTPs is often linked with voluntary unloading, altered motor patterns, and protective movement adaptations that reduce stress on affected tissues (Dommerholt et al., 2019; Tidball, 2017).

### 4.4 Structural and metabolic (energy crisis/integrated hypothesis) models

Structural and imaging studies have identified increased tissue stiffness, taut bands, and localized architectural alterations within MTP regions, supporting the presence of measurable biomechanical changes (Shah et al., 2015; Sadria et al., 2016; Ge et al., 2025). Histological and physiological findings, including mitochondrial stress, oxidative signaling, and sarcomere disorganization, have been interpreted as indicators of localized metabolic strain and tissue remodeling (Chapman et al., 2016). These structural characteristics are frequently associated with altered force transmission, reduced elasticity, and mechanical sensitivity, which may contribute to both functional limitation and persistent nociceptive input.

### 4.5 Latent trigger points: subclinical nociceptive and motor phenomena

In addition to clinically active trigger points, the literature consistently distinguishes between active and latent MTPs, with latent points defined as palpable taut bands that do not produce spontaneous pain but remain mechanically sensitive and capable of eliciting referred pain upon stimulation (Ge et al., 2009; Ge and Arendt-Nielsen, 2011; Fernández-de-las-Peñas and Dommerholt, 2014). Experimental studies have demonstrated that latent MTPs are not physiologically inactive; rather, they exhibit altered motor control, increased muscle fatigability, and heightened sensitivity to mechanical and nociceptive stimuli (Ge et al., 2009).

Ge et al. (2009) reported that muscles harboring latent MTPs demonstrate reduced endurance and impaired activation patterns compared to non-MTP muscle tissue, suggesting that latent trigger points may influence neuromuscular function even in the absence of overt pain. Further work by Ge and Arendt-Nielsen (2011) indicated that latent MTPs can act as persistent peripheral nociceptive sources capable of contributing to central sensitization under sustained stimulation or mechanical stress. These findings support the conceptualization of MTPs as dynamic physiological entities that may exist along a continuum of activity rather than as strictly binary active or inactive structures.

Clinical syntheses have similarly emphasized the prevalence and relevance of latent MTPs in both symptomatic and asymptomatic populations (Fernández-de-las-Peñas and Dommerholt, 2014). Their presence has been associated with altered movement patterns, reduced range of motion, and increased susceptibility to the development of active trigger points under conditions of sustained mechanical load or repetitive strain. This continuum-based perspective complicates purely symptom-driven diagnostic models and suggests that the absence of spontaneous pain does not necessarily indicate the absence of underlying physiological activity within the trigger point region.

### 4.6 Skeptical and construct validity perspectives

In parallel with literature on prevailing models, a distinct body of literature has raised concerns regarding the construct validity, diagnostic reliability, and empirical grounding of MTPs as discrete pathophysiological entities. Rather than disputing the presence of localized tenderness, referred pain, or palpable taut bands, skeptical perspectives primarily question whether these clinical findings reliably correspond to a unique and biologically distinct tissue abnormality with clearly defined diagnostic boundaries (Quintner et al., 2015; Wilke et al., 2018).

A central issue identified in this literature is the variability and inconsistency of diagnostic criteria. Multiple reviews have noted that trigger point identification relies heavily on manual palpation and examiner interpretation, with substantial variability in inter-examiner reliability across studies. While clinical clusters such as taut band, hypersensitive spot, and referred pain are commonly used, their reproducibility and specificity remain debated, particularly in blinded or standardized assessments (Barbero et al., 2019; Quintner et al., 2015). The absence of a universally accepted reference standard further complicates efforts to validate MTP diagnosis in experimental settings.

Concerns have also been raised regarding potential circular diagnostic reasoning within traditional MTP frameworks. Specifically, the presence of pain may be used to justify the identification of an active trigger point, while the absence of spontaneous pain is attributed to a latent trigger point, thereby reducing falsifiability of the construct and allowing broad interpretive flexibility (Quintner et al., 2015). From a construct validity standpoint, this creates difficulty in establishing clear operational boundaries between pathological entities and clinically descriptive phenomena.

Methodological critiques extend to proposed objective correlates of MTPs, including biochemical, electrophysiological, and imaging findings. Although elevated concentrations of inflammatory mediators, spontaneous electrical activity (SEA), and localized stiffness have been documented in trigger point regions, skeptical analyses note that such findings may not be entirely specific to MTPs and may also occur in other contexts of muscle stress, inflammation, or peripheral nerve sensitization (Quintner et al., 2015; Wilke et al., 2018). Similarly, while emerging imaging modalities such as ultrasound and elastography show promise in identifying tissue heterogeneity, limitations in study design, comparison standards, and diagnostic criteria currently restrict their use as definitive validation tools (Dommerholt et al., 2019).

Treatment-based validity has also been discussed within skeptical frameworks. Some authors argue that interventions targeting presumed trigger points, including needling and manual therapies, may produce clinical improvements that are not necessarily specific to a localized pathological entity, but instead reflect non-specific analgesic, neurophysiological, or contextual treatment effects (Quintner et al., 2015). Importantly, these critiques do not deny the clinical reality of patient-reported pain, functional limitation, or localized muscle sensitivity, but rather challenge the assumption that such findings must originate from a singular, discrete pathological lesion.

Despite these critiques, even skeptical perspectives acknowledge that patients frequently present with reproducible regions of localized tenderness, altered sensitivity, and motor dysfunction that are clinically meaningful. As such, the ongoing debate is less centered on whether observable phenomena exist, and more on how they should be conceptualized, operationalized, and biologically interpreted within a coherent theoretical framework. This tension between clinical reproducibility and physiological ambiguity highlights a key unresolved issue in the MTP literature: the field possesses substantial descriptive and physiological data, yet lacks a universally accepted integrative model that fully accounts for the formation, persistence, variability, and clinical behavior of trigger points across contexts.

Beyond theoretical disagreement, an additional source of fragmentation in the MTP literature may be methodological, with prevailing models having been shaped by specific assessment tools available to investigators, each of which captures only a partial dimension of the phenomenon.

### 4.7 Assessment constraints and their potential influence on prevailing model development

A major source of conceptual disagreement in the MTP literature may be that prevailing models have often been shaped by the dominant assessment tools available at a given time. Historically, diagnosis has relied primarily on palpation and symptom reproduction, which remain clinically important but are inherently examiner-dependent and limited in sensitivity to subtle tissue variation (Ballyns et al., 2012; Shah et al., 2015). Methods such as pressure algometry, electromyography, ultrasound, elastography, Doppler imaging, and localized biochemical sampling have each added objective information, but each preferentially captures only one dimension of the phenomenon (mechanical sensitivity, electrical activity, tissue stiffness, vascular behavior, or biochemical milieu) (Sikdar et al., 2009). As a result, differences across prevailing models may reflect not only biological complexity, but also the selective biases of the measurement approaches used to study MTPs.

**Figure 1:**
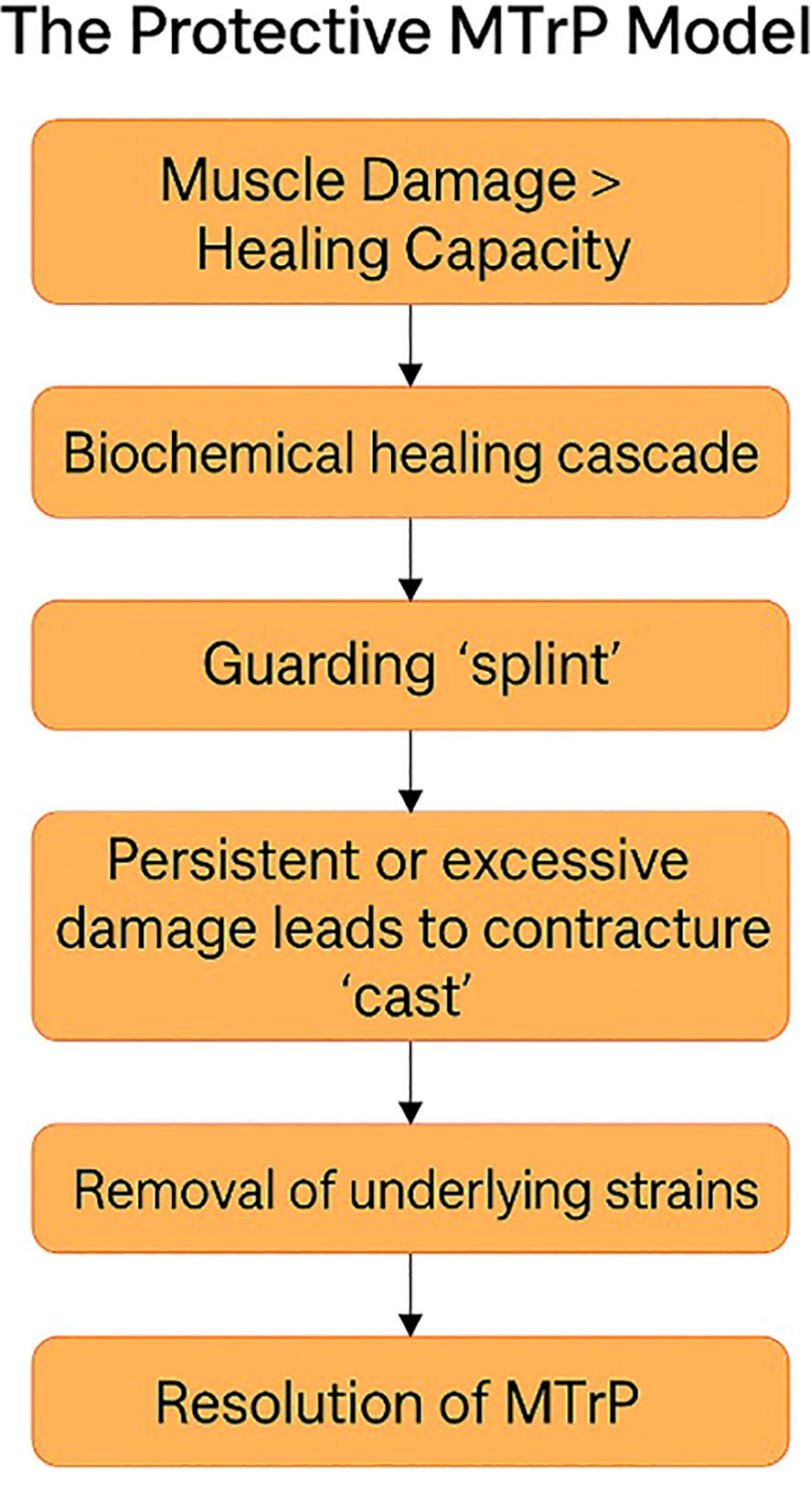
Assessment-driven fragmentation of MTP models different assessment methods capture different dimensions of MTPs and may therefore favor fragmented explanatory models. The Protective Model integrates these partial perspectives within a multimodal framework.

In this context, recent ultrasound work has been especially informative in showing that even ostensibly objective elasticity measures are constrained by methodological factors such as tissue depth, local structural heterogeneity, and dissipation of externally applied force through surrounding tissue (Tsai et al., 2024). These limitations suggest that no single assessment modality fully captures MTP biology. Rather, a useful predictive framework should generate converging hypotheses across multiple modalities while remaining compatible with the partial, scale-dependent nature of existing measurement techniques. **Figure 1** illustrates how reliance on isolated assessment domains may have shaped fragmented prevailing models and how an integrative framework may help reconcile these partial perspectives.

### 4.8 Integration

Collectively, prevailing models portray MTPs as biologically active regions characterized by sustained neuromuscular activity, localized biochemical signaling, sensory sensitization, and measurable structural alterations, each of which can be assessed to some extent but with important limitations. However, these mechanisms are most often studied within isolated theoretical domains, resulting in fragmented explanations that emphasize neuromuscular dysfunction, metabolic stress, inflammatory activity, or nociceptive amplification independently. While each framework provides valuable insight into specific aspects of MTP physiology, a unified model that fully accounts for their formation, persistence, variability, and clinical behavior remains incomplete in the current literature. **Table 2** summarizes the prevailing models of MTPs described in the literature.

**Table 2:** Models of MTPs in current literature.

Model	Core mechanism	Key authors	Representative quote from source
Peripheral and Central Sensitization Models	Ongoing nociceptive input from MTPs contributes to peripheral sensitization and CNS amplification	Fernández-de-las-Peñas et al. (2007); Ge et al. (2009); Ge and Arendt-Nielsen (2011)	“The referred pain pattern associated with myofascial trigger points is generated and maintained by central sensitization mechanisms.” – Fernández-de-las-Peñas et al. (2007)
Integrated Hypothesis/Energy Crisis Model	Sustained sarcomere contraction leads to ischemia, hypoxia, ATP depletion, and persistent contracture	Travell and Simons; Dommerholt et al. (2019); Shah et al. (2015)	“The sustained sarcomere contraction reduces blood flow, resulting in hypoxia and a decreased supply of ATP … resulting in a localized energy crisis.” – Dommerholt et al. (2019)
Neuromuscular Dysfunction and Motor Endplate Models	Abnormal endplate activity, SEA, and altered motor unit recruitment sustain localized contraction	Jafri (2014); Shah et al. (2015); Ge et al. (2025)	“Motor endplate noise and spontaneous electrical activity have been frequently observed in MTrPs … indicating a neuromuscular disorder in the affected region.” – Jafri (2014)
Biochemical and Neuroimmune Models	Elevated cytokines, neuropeptides, and inflammatory mediators drive local sensitization and nociception	Shah et al. (2015); Sadria et al. (2016); Lv and Yin (2024); Hadizadeh et al. (2025)	“Biochemical milieu of active MTrPs contains significantly elevated levels of proinflammatory and nociceptive substances including bradykinin, substance P, TNF-α, IL-1β, IL-6.” – Shah et al. (2015)
Active–Latent Trigger Point Continuum Models	MTPs exist along a physiological continuum, with latent points showing altered motor control and nociceptive sensitivity despite absence of spontaneous pain	Ge et al. (2009); Ge and Arendt-Nielsen (2011); Fernández-de-las-Peñas and Dommerholt (2014); Bron and Dommerholt (2012)	“Latent myofascial trigger points may contribute to motor dysfunction and serve as persistent nociceptive sources even in the absence of spontaneous pain.” – Ge and Arendt-Nielsen (2011)
Skeptical and Construct Validity Perspectives	Questioning diagnostic reliability, specificity, biomarkers, and the existence of MTPs as discrete pathological entities	Quintner et al. (2015); Wilke et al. (2018); Barbero et al. (2019)	“The diagnostic criteria for trigger points lack specificity and reliability, and there is no consistent imaging or histopathological correlate that defines an MTrP.” – Quintnet et al. (2015)

## 5 Conceptualization of the protective model of myofascial trigger points

Traditionally, MTPs have been interpreted as pathological manifestations of aberrant neuromuscular activity, metabolic disruption, or central sensitization. Although these processes are well documented, they are often described in isolation and do not provide a unified physiological framework that is predictive, biologically grounded, and falsifiable. Few models explicitly situate the emergence of MTPs within systems-level tissue regulation under sustained mechanical strain. The Protective Model addresses this gap.

The Protective Model conceptualizes MTPs as adaptive sentinel responses, as previously defined, that emerge when cumulative tissue strain surpasses local muscle repair capacity. Within this framework, MTPs are understood not as spontaneous pathological lesions but as patterned physiological responses to unresolved strain–repair mismatch. The neuromuscular activity, biochemical signaling, tissue stiffness, and nociceptive amplification described in the literature are therefore understood as coordinated components of an adaptive sentinel response that limits further strain under conditions of persistent tissue stress.

Evidence supporting this reframing is present in prior work linking MTP formation to regions of biomechanical dysfunction. Fernández-de-las-Peñas et al. (2004) demonstrated a strong association between cervical joint dysfunction and MTP presence, with symptom improvement often greater following treatment of the joint itself. These findings support the view that MTPs arise in regions experiencing altered mechanical loading and reflect physiological responses to persistent strain rather than isolated tissue abnormalities.

### 5.1 Model development and theoretical framework construction

This model was developed through structured conceptual integration of recurring physiological themes within the MTP literature, including neuromuscular activity, biochemical signaling, tissue stiffness, and nociceptive amplification. Rather than functioning as a systematic review, the present work synthesizes convergent empirical findings across physiological domains to construct an integrative explanatory framework. Accordingly, the model prioritizes physiological coherence, biological plausibility, and falsifiability as primary criteria for framework construction over exhaustive aggregation of individual studies.

Throughout the explanation of the Protective Model, the term “protective” refers to emergent biological responses that limit further mechanical strain under conditions of sustained tissue stress, without implying conscious intent, teleological regulation, or goal-directed behavior.

### 5.2 Foundational principle: strain-repair mismatch

Skeletal muscle undergoes microscopic sarcomere disruption during normal use, with greater structural perturbation occurring during repetitive loading, eccentric contraction, and compensatory recruitment patterns (Jafri, 2014; Bron and Dommerholt, 2012; Jin et al., 2020). Under typical conditions, microdamage is resolved through protein synthesis, mitochondrial restoration, and extracellular matrix remodeling during recovery periods. When mechanical demand persistently exceeds the rate of cellular repair and metabolic restoration, microdamage accumulates faster than physiological recovery processes can resolve.

Within the Protective Model, this imbalance constitutes a strain–repair mismatch. The adaptive sentinel response emerges in regions where localized structural compromise increases susceptibility to further mechanical deformation. Reduced tensile continuity within affected sarcomeres alters load distribution across the fiber, increasing mechanical vulnerability under continued strain. Clinical observations that mechanical overload and compensatory recruitment patterns contribute to MTP development are consistent with this interpretation (Barbero et al2019).

Repair capacity is not determined solely by local tissue properties. Systemic factors known to influence recovery processes, including sleep quality, metabolic health, psychosocial stress, and overall physiological load, may modulate regenerative efficiency. Accordingly, strain–repair mismatch may arise from increased mechanical demand, diminished repair capacity, or both. This interpretation remains physiologically specific while also accommodating broader biopsychosocial influences on musculoskeletal adaptation.

Within this framework, strain–repair mismatch may emerge after a single sufficiently damaging mechanical event, through repeated submaximal loading, or through the cumulative effects of persistent demand occurring in the setting of inadequate recovery.

### 5.3 Local contractile component of the adaptive sentinel response

In regions of structural compromise, nociceptive input and altered motor endplate regulation may initiate localized contractile activity consistent with a protective guarding response (Barbero et al., 2019; Ge et al., 2009; Gerwin, 2023; Shah et al., 2015). If incomplete repair persists, sustained sarcomere shortening may develop, producing localized contracture. This contracture generates a mechanically stiffened zone surrounding compromised fibers.

Over time, repeated strain under unresolved strain–repair mismatch may promote extracellular matrix remodeling and progressive stiffening within the affected region (Forcina et al., 2020; Ge et al., 2025). Within the Protective Model, this localized stiffening is interpreted as a structural component of the adaptive sentinel response that reduces deformation and shear stress across mechanically vulnerable fibers.

Spontaneous electrical activity observed within MTP regions is consistent with persistent motor endplate dysregulation under sustained contracture (Ge et al., 2009; Jafri, 2014; Holanda et al., 2015). Localized shortening and increased stiffness may reduce mechanical propagation of microtearing, thereby limiting additional structural disruption within compromised tissue.

Experimental models demonstrating spontaneous resolution of trigger point–like contractures following removal of sustained loading further support the interpretation that such contractures are contingent on ongoing strain–repair mismatch rather than permanent pathological change (Itoh et al., 2004).

### 5.4 Neuroimmune component of the adaptive sentinel response

Biochemical analyses consistently reveal elevated concentrations of Substance P, calcitonin gene-related peptide, bradykinin, IL-1β, IL-6, and TNF-α within MTP regions (Forcina et al., 2020; Ge et al., 2025; Hadizadeh et al., 2025; Jafri, 2014; Shah et al., 2015; Sadria et al., 2016; Tidball, 2017). These mediators are associated with nociceptor sensitization, immune activation, vasomodulation, and extracellular matrix remodeling.

Within the Protective Model, this biochemical milieu is interpreted as consistent with a localized stress-associated microenvironment observed in tissue repair processes. Hypoxia and reduced perfusion, frequently described as dysfunction, are also predictable consequences of sustained contracture and parallel known patterns of angiogenic signaling during muscle healing (Jin et al., 2020; Holanda et al., 2015).

### 5.5 Sensory component of the adaptive sentinel response

Peripheral sensitization within MTP regions results in reduced nociceptor thresholds and amplified pain signaling (Fernández-de-las-Peñas et al., 2007; Shah et al., 2015). Repeated nociceptive input may facilitate central sensitization through spinal and supraspinal amplification pathways, contributing to referred pain and expanded zones of hyperalgesia (Dommerholt et al., 2019; Suresh et al., 2021).

Within the Protective Model, this sensory amplification is interpreted as a load-modulating component of the adaptive sentinel response, as heightened nociceptive sensitivity is associated with voluntary unloading, altered recruitment patterns, and reduced mechanical exposure of compromised tissue.

### 5.6 Structural remodeling component of the adaptive sentinel response

Elastographic and ultrasonographic studies consistently demonstrate reduced compliance and increased stiffness in MTP-containing tissue (Ballyns et al., 2012; Silva et al., 2025; Wilke et al, 2018). Microscopic analyses reveal sarcomere disorganization, mitochondrial stress, and oxidative markers consistent with altered metabolic demand (Shah et al., 2015; Sadria et al., 2016).

Within the Protective Model, localized stiffening is interpreted as a redistribution of mechanical load away from compromised fibers. This phenomenon parallels established physiological responses such as post-injury muscle guarding and externally applied mechanical stabilization strategies that reduce strain on vulnerable tissue.

### 5.7 Trigger points as adaptive sentinel response

Taken together, the neuromuscular, biochemical, sensory, and structural features associated with MTPs are consistent with a coordinated adaptive sentinel response emerging under persistent strain–repair mismatch. Chronicity reflects continued mechanical demand or insufficient repair capacity rather than intrinsic dysfunction of the MTP itself.

In this framework, MTPs are not biologically distinct entities categorized as latent or active but represent varying magnitudes of expression within a single structural and physiological pattern. Pain emerges when strain–repair mismatch reaches a threshold sufficient to generate sustained nociceptive signaling. Variability in classification reliability may therefore reflect gradations in expression rather than fundamentally separate pathophysiological states (Barbero et al., 2019).

**Figure 2:**
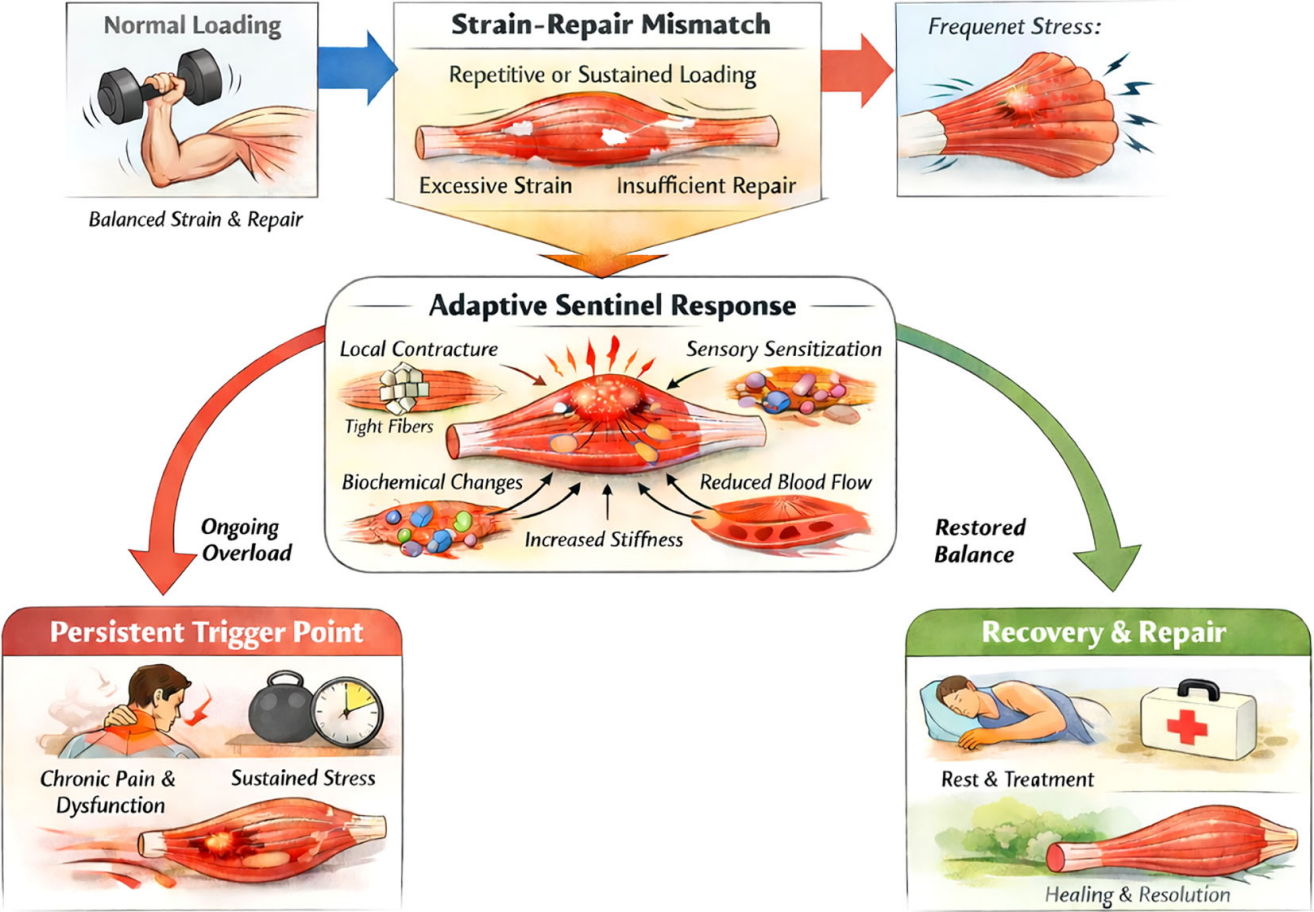
Systems-level skeletal muscle depiction of the protective model of myofascial trigger point formation. Illustration of the Protective Model showing how strain–repair mismatch may trigger a localized adaptive sentinel response and lead to either persistent MTP expression under continued overload or normalization when strain–repair balance is restored.

**Figure 3:**
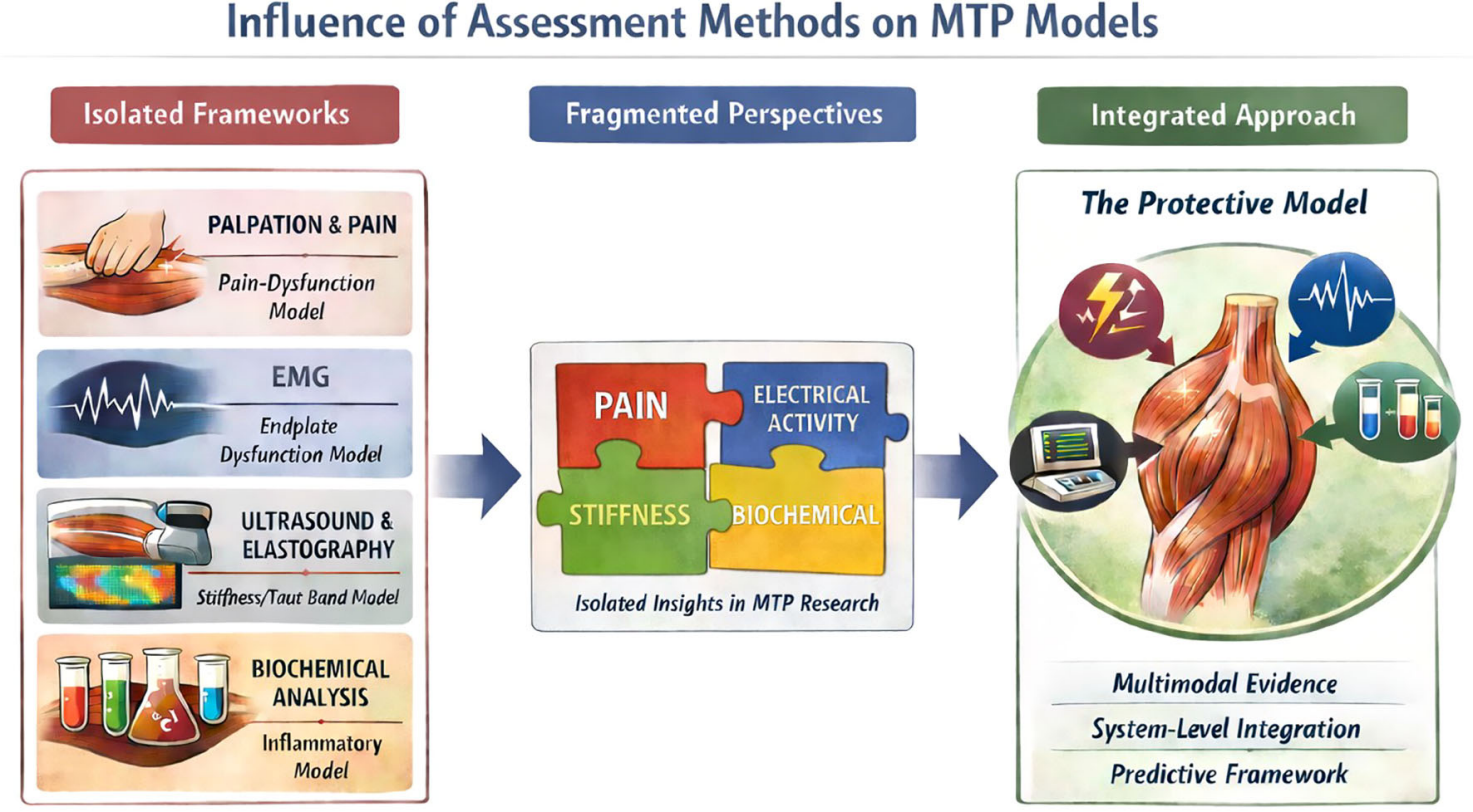
Simplified stepwise schematic of the protective model Illustrative stepwise representation of the proposed pathway; individual cases may vary in sequence or magnitude of expression.

Rather than viewing MTPs as pathological remnants, the Protective Model interprets them as structured physiological responses consistent with load-regulation patterns under persistent musculoskeletal stress, as illustrated in **Figure 2**. **Figure 3** provides a simplified stepwise schematic of this proposed pathway, depicting how strain–repair mismatch may progress through biochemical signaling and protective guarding toward contracture, and how resolution is expected when underlying mechanical strain is removed.

## 6 Predictions and testable hypothesis

**Figure 4:**
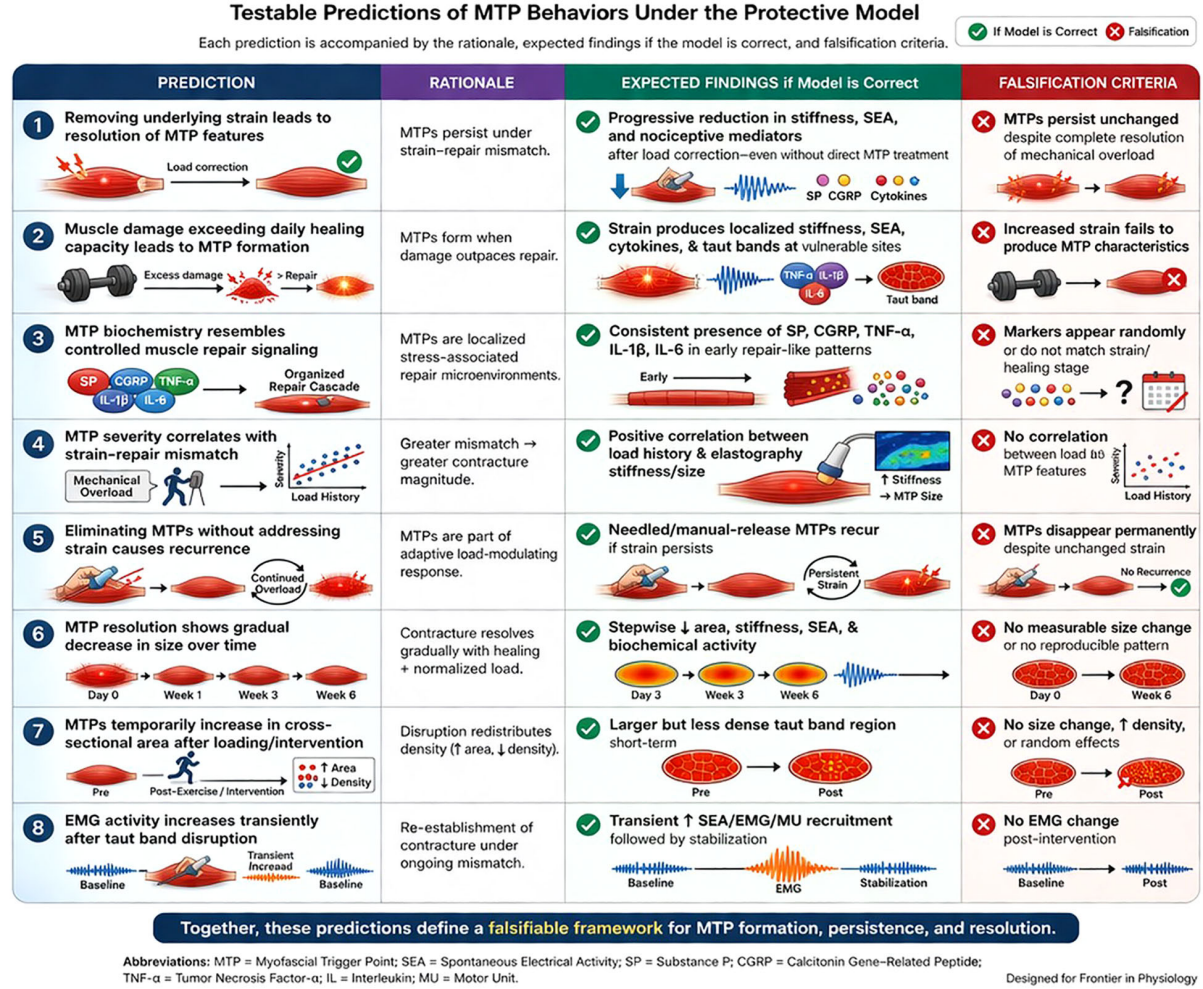
Visual overview of the protective model predictions Illustration of the principal experimental predictions summarized in **Table 3**, including MTP resolution with strain correction, formation under excessive strain–repair mismatch, coordinated biochemical signaling, load-related variation in MTP severity, recurrence under persistent load, progressive reduction during recovery, transient post-intervention redistribution, and short-term electrophysiological changes after taut band disruption.

The Protective Model predicts that MTPs emerge when cumulative local muscular strain exceeds the rate of physiological tissue repair, resulting in a persistent strain–repair mismatch. This mismatch is proposed to initiate an adaptive sentinel response characterized by coordinated neuromuscular, biochemical, sensory, and structural changes that reduce loading of compromised fibers. From this framework, a set of biologically grounded and falsifiable predictions follows, distinguishing the Protective Model from prevailing pathological or dysfunction-based theories. **Table 3** summarizes the principal testable predictions generated by the Protective Model and their corresponding falsification criteria, and **Figure 4** provides a visual overview of these proposed experimental domains.

**Table 3:** Testable predictions of MTP behaviors under the protective model.

Prediction	Rationale	Expected findings if model is correct	Falsification criteria
1. Removing underlying strain leads to resolution of MTP features	MTPs persist under strain-repair mismatch.	Progressive reduction in stiffness, SEA, and nociceptive mediators after load correction—even without direct MTP treatment	MTPs persist unchanged despite complete resolution of mechanical overload
2. Muscle damage exceeding daily healing capacity leads to MTP formation	MTPs form when damage outpaces repair	Experimentally induced strain produces localized stiffness, SEA, cytokine elevation, and taut bands at mechanically vulnerable sites	Increased strain fails to produce MTP characteristics
3. MTP biochemistry resembles controlled muscle repair signaling rather than nonspecific inflammatory activity	Protective model frames MTPs as localized stress-associated repair microenvironments	Consistent presence of SP, CGRP, TNF-α, IL-1β, IL-6 in patterns resembling normal muscle repair cascades	Biochemical markers appear randomly or do not correspond to strain or healing stage
4. MTP severity correlates with strain–repair mismatch	Greater strain–repair mismatch is predicted to be associated with increased contracture magnitude	Positive correlation between mechanical overload history and elastography stiffness and size of MTP	No observable correlation between load and MTP characteristics
5. Eliminating MTPs without addressing strain causes recurrence	MTPs function as components of the adaptive sentinel response associated with load modulation; removing MTP features without correcting underlying mechanical load may allow strain–repair mismatch to persist or recur	Needled/manual-release MTPs recur if underlying strain persists	MTPs disappear permanently despite unchanged mechanical strain
6. MTP resolution, with or without intervention, will show a gradual decrease in size as healing proceeds	Contracture is predicted to gradually resolve as tissue repair progresses and mechanical load normalizes	Stepwise reduction in area, stiffness, SEA, and biochemical activity overtime	No measurable size change or no reproducible post-loading redistribution pattern
7. MTPs temporarily increase in cross-sectional area after mechanical loading or intervention	Disruption redistributes contracture density while decreasing stiffness	Larger but less dense taut band region post-exercise/intervention	No measurable size change; contradictory density increase; random effects
8. EMG activity increases transiently after taut band disruption	Neuromuscular activity may transiently increase, consistent with re-establishment of localized contracture under continued strain–repair mismatch	Transient increase in SEA, EMG, and/or MU recruitment followed by stabilization	No EMG change

### 6.1 Practical clinical examples applying the protective model

A common example is repetitive deadlifting performed primarily through lumbar extension rather than coordinated abdominal stabilization and lower-extremity loading. Under these conditions, the lumbar paraspinal musculature may be exposed to mechanical demand that exceeds local repair capacity, whether through repeated submaximal loading, excessive loading intensity, or the cumulative effects of inadequate recovery and compensatory recruitment. Within the Protective Model, this strain–repair mismatch would increase the likelihood of adaptive sentinel response emergence in the involved musculature, expressed through localized stiffness, contracture, nociceptive sensitivity, and altered motor behavior. In this context, MTP formation would not be interpreted as an isolated pathological lesion, but as a physiological response arising in tissue exposed to sustained overload without sufficient restoration.

A similar pattern may occur in the masticatory system during habitual teeth clenching or sustained jaw tension. Prolonged low-level contraction of the masseter, temporalis, and related musculature may subject these tissues to repeated metabolic and mechanical stress even in the absence of overt injury. If this demand persists beyond daily local repair capacity, the Protective Model predicts that adaptive sentinel responses may emerge within these muscles, contributing to localized tenderness, stiffness, and referred pain. This example illustrates that strain–repair mismatch need not depend on high-force injury alone, but may also develop through sustained submaximal loading maintained over time.

Joint dysfunction may also interact bidirectionally with MTP formation within this framework. Altered joint mechanics may redistribute force to surrounding musculature, typically tasked with maintaining joint alignment, to perform compensatory recruitment and localized tissue strain in muscles required to stabilize or offload the dysfunctional region. Under persistent conditions, this may increase the likelihood of MTP formation in adjacent tissue. Conversely, once present, MTP-associated stiffness, pain, and altered recruitment may further disrupt movement patterns and load distribution, increasing stress across the involved joint and contributing to joint pain or persistent dysfunction. Within the Protective Model, this reciprocal relationship helps explain how articular dysfunction and myofascial findings may perpetuate one another through ongoing strain–repair mismatch and biomechanical compensation.

## 7 Discussion: comparing prevailing models to the protective model of MTPs

In contrast to prevailing explanatory frameworks, the proposed Protective Model conceptualizes MTPs not as isolated dysfunctions, but as biologically mediated responses that emerge in the context of sustained mechanical or physiological strain. Rather than rejecting existing findings, the Protective Model reinterprets the mechanisms described in the literature within a systems-level framework centered on strain–repair mismatch and adaptive sentinel response. Across domains, prevailing interpretations emphasize dysfunction within isolated physiological systems, whereas the Protective Model proposes that these same observations may represent coordinated expressions of a single strain–repair mismatch process.

### 7.1 Neuromuscular dysfunction and motor endplate models

Neuromuscular dysfunction models describe MTPs as regions of abnormal motor endplate activity characterized by spontaneous electrical activity (SEA), endplate noise, and sustained sarcomere contraction. Jafri (2014) noted that “Motor endplate noise and spontaneous electrical activity have been frequently observed in MTrPs … indicating a neuromuscular disorder in the affected region.” Within this framework, persistent contracture and altered motor unit recruitment are interpreted as indicators of localized neuromuscular pathology.

The Protective Model acknowledges these electrophysiological findings but recontextualizes them as regulated motor output adjustments associated with tissue stress rather than primary neuromuscular failure. Sustained localized contraction may contribute to mechanical stabilization of the affected region and reduced mechanical deformation of compromised muscle fibers. From this perspective, persistent contracture is interpreted not solely as maladaptive neuromuscular dysfunction, but as a potential component of the adaptive sentinel response emerging under sustained strain–repair mismatch.

### 7.2 Biochemical and neuroimmune models of MTPs

Biochemical and neuroimmune models emphasize the presence of elevated inflammatory mediators, including Substance P, bradykinin, CGRP, TNF-α, IL-1β, and IL-6, within MTP regions (Shah et al., 2015; Lv and Yin, 2024). These findings are typically interpreted as evidence of localized sensitization, inflammation, and dysfunctional tissue physiology contributing to pain amplification and persistent nociceptive signaling.

The Protective Model does not dispute the presence of these mediators but interprets them within the broader context of coordinated tissue stress and repair responses. Rather than signifying unregulated inflammation, their presence may reflect stress-associated signaling pathways consistent with coordinated muscle repair and remodeling processes described in muscle physiology literature (Shah et al., 2015; Forcina et al., 2020; Tidball, 2017; Ge et al., 2025). Consequently, the biochemical milieu observed in MTPs may represent an active physiological response to sustained microtrauma and strain–repair mismatch rather than isolated pathological dysfunction.

### 7.3 Peripheral and central sensitization models

Sensitization-based models conceptualize MTPs as persistent nociceptive sources capable of driving both peripheral and central sensitization. Fernández-de-las-Peñas et al. (2007) proposed that “The referred pain pattern associated with myofascial trigger points is generated and maintained by central sensitization mechanisms,” while Ge et al. (2009) and Ge and Arendt-Nielsen (2011) demonstrated that latent MTPs can contribute to ongoing nociceptive input and sensory amplification.

The Protective Model acknowledges the role of sensitization but positions MTPs as upstream contributors within a broader physiological context rather than purely downstream manifestations of central nervous system amplification. Within this framework, increasing strain–repair mismatch may elevate nociceptive signaling to levels associated with voluntary unloading and altered motor behavior. Heightened nociceptor sensitivity may therefore contribute to behavioral unloading and modified recruitment patterns that reduce mechanical exposure of stressed tissue, aligning with the adaptive sentinel response construct.

### 7.4 Structural and metabolic (energy crisis/integrated hypothesis) models

The Integrated Hypothesis, originating from the work of Travell and Simons and expanded by Dommerholt et al. (2019), describes MTPs as regions of sustained sarcomere contraction leading to ischemia, hypoxia, and ATP depletion. As noted by Dommerholt et al. (2019), “The sustained sarcomere contraction reduces blood flow, resulting in hypoxia and decreased supply of ATP … resulting in localized energy crisis.” Within this model, persistent contraction is framed as a self-perpetuating pathological cycle driven by metabolic collapse.

The Protective Model incorporates these metabolic findings but reinterprets them within the framework of strain–repair mismatch and adaptive sentinel response. Localized hypoxia, reduced perfusion, and metabolic stress may emerge secondary to sustained contracture that develops in mechanically compromised tissue. In this interpretation, metabolic alterations described in the energy crisis model may represent downstream physiological consequences of sustained load modulation rather than primary pathological drivers. This perspective maintains consistency with observed patterns of post-injury muscle guarding and reduced tissue loading following structural compromise.

### 7.5 Active-latent trigger point continuum models

Continuum models distinguish between active and latent MTPs, with latent points demonstrating altered motor control, increased fatigability, and mechanical sensitivity despite the absence of spontaneous pain (Ge et al., 2009; Ge and Arendt-Nielsen, 2011; Fernández-de-las-Peñas and Dommerholt, 2014). These findings suggest that MTPs may exist along a dynamic physiological spectrum rather than as discrete binary entities.

The Protective Model strongly aligns with this continuum perspective, proposing that latent trigger points represent lower-magnitude expressions of the adaptive sentinel response that intensify as strain–repair mismatch persists. Under this paradigm, increases in MTP size, sensitivity, and clinical impact reflect ongoing unresolved mechanical load rather than sudden pathological onset. This interpretation provides a systems-level physiological explanation for gradual progression, recurrence, and context-dependent variability frequently observed in clinical populations.

### 7.6 Skeptical and construct validity perspectives

Skeptical perspectives question the diagnostic reliability, specificity, and construct validity of MTPs as discrete pathological entities (Quintner et al., 2015; Wilke et al., 2018). Concerns include variability in palpation reliability, lack of universally accepted biomarkers, and potential circular reasoning inherent in active versus latent classifications.

The Protective Model acknowledges these critiques and partially addresses them by reframing MTPs not as fixed pathological lesions but as context-dependent physiological responses emerging under strain–repair mismatch. This interpretation allows for variability in clinical presentation, gradual development, and fluctuating symptom expression without requiring rigid binary diagnostic categorization. Additionally, anchoring investigation to measurable features such as taut bands, spontaneous electrical activity, and elastographic stiffness, while interpreting symptom variability as downstream effects, may support the development of more reproducible research and diagnostic frameworks.

### 7.7 Summary

Across domains, prevailing interpretations emphasize dysfunction within isolated physiological systems, whereas the Protective Model proposes that these same observations may represent coordinated expressions of an adaptive sentinel response arising from strain–repair mismatch.

Importantly, not all regions subjected to mechanical overload appear to develop clinically detectable MTPs. Variability in tissue resilience, repair efficiency, habitual loading patterns, and neuromuscular recruitment likely modulates the expression of adaptive sentinel responses across individuals and anatomical regions.

Accordingly, the Protective Model does not assume universal MTP formation under overload; rather, it proposes that adaptive sentinel responses emerge as a stochastic physiological process when local strain–repair mismatch exceeds tissue-specific and systemic thresholds, and may remain subclinical or latent when these thresholds are only partially exceeded.

## 8 Explanatory gaps in prevailing models and how the protective model addresses them

Despite substantial advances in the characterization of MTPs, prevailing models remain largely domain-specific, emphasizing neuromuscular dysfunction, metabolic stress, sensitization, or biochemical alterations in relative isolation. While each framework accounts for important physiological findings, several recurrent clinical and experimental observations remain only partially explained within single-mechanism models. This fragmentation has contributed to ongoing conceptual ambiguity regarding the formation, persistence, variability, and clinical behavior of MTPs.

### 8.1 Gap 1: recurrence of MTPs following localized treatment

A frequently reported clinical phenomenon is the recurrence of MTPs after interventions targeting the local tissue, including dry needling, manual therapy, and injection-based approaches. While such treatments often produce short-term analgesic and functional improvements, long-term resolution is inconsistent when underlying mechanical or functional stressors remain unaddressed (Dommerholt et al., 2019; Bron and Dommerholt, 2012).

Prevailing models, particularly energy crisis and neuromuscular dysfunction frameworks, primarily interpret MTPs as localized pathological entities. This interpretation does not fully account for the tendency of trigger points to re-emerge in the same anatomical regions despite apparent local deactivation.

The Protective Model addresses this gap by conceptualizing MTPs as expressions of adaptive sentinel response arising from unresolved strain–repair mismatch rather than isolated lesions. Within this framework, recurrence reflects persistence of the underlying mechanical demand or impaired repair capacity that initially precipitated the adaptive sentinel response.

### 8.2 Gap 2: active-latent continuum and subclinical physiological activity

The consistent identification of latent MTPs, defined as palpable taut bands that do not produce spontaneous pain yet exhibit mechanical sensitivity and altered motor function, presents a challenge to strictly symptom-driven or pathology-centered models (Ge et al., 2009; Ge et al., 2012; Fernández-de-las-Peñas and Dommerholt, 2014).

Sensitization models explain nociceptive amplification but do not fully clarify why physiologically active trigger points can persist in asymptomatic states. Similarly, structural and metabolic models focus on localized dysfunction without explicitly addressing graded progression across a continuum.

The Protective Model provides a coherent explanation by proposing that latent MTPs represent lower-magnitude expressions of the adaptive sentinel response that intensify as strain–repair mismatch persists. This continuum framework aligns with empirical findings demonstrating altered endurance, motor control, and nociceptive sensitivity in muscles harboring latent MTPs (Ge et al., 2009).

### 8.3 Gap 3: presence of SEA without definitive structural lesion

Electrophysiological studies consistently report spontaneous electrical activity and endplate noise in MTP regions (Jafri, 2014; Shah et al., 2015), yet clearly defined histopathological lesions are not uniformly identified. This creates tension within models that frame MTPs strictly as localized neuromuscular pathology.

The Protective Model reconciles this discrepancy by interpreting spontaneous electrical activity as a manifestation of context-dependent motor output associated with sustained contracture within the adaptive sentinel response rather than evidence of primary neuromuscular failure. Sustained localized activation may contribute to mechanical stabilization and reduced deformation of structurally stressed tissue, without requiring the presence of a discrete anatomical lesion.

### 8.4 Gap 4: biochemical activity without a discrete pathological entity

Biochemical analyses have demonstrated elevated concentrations of inflammatory mediators, neuropeptides, and nociceptive substances within MTP regions (Shah et al., 2015; Sadria et al., 2016; Lv and Yin, 2024). However, these findings are not entirely specific to MTPs and may also occur in broader contexts of muscle stress, inflammation, and tissue repair, complicating interpretations of MTPs as discrete pathological lesions (Quintner et al., 2015).

Rather than signifying unregulated inflammation, these biochemical patterns may be more consistent with stress-associated neuroimmune signaling observed in coordinated muscle repair processes (Forcina et al., 2020; Tidball, 2017; Ge et al., 2025). The Protective Model integrates these findings by framing the biochemical milieu as a component of the adaptive sentinel response emerging under strain–repair mismatch.

### 8.5 Gap 5: predictable anatomical localization and mechanical load concentration

Clinically, MTPs are frequently identified in muscles subjected to sustained postural load, repetitive use, or chronic mechanical stress, such as the upper trapezius, lumbar paraspinals, and masticatory musculature (Bron and Dommerholt, 2012; Fernández-de-las-Peñas and Dommerholt, 2014; Shah et al., 2015). While prevailing models acknowledge localized dysfunction within affected tissue, they do not fully explain the consistent association between trigger point development and regions of prolonged mechanical demand.

The Protective Model directly incorporates mechanical load distribution as a primary initiating factor, proposing that regions subjected to sustained strain exceeding local repair capacity are more likely to develop an adaptive sentinel response characterized by contracture, biochemical signaling, and sensitization. This systems-level integration provides a physiological explanation for both anatomical localization and recurrence patterns observed in clinical populations.

### 8.6 Gap 6: construct validity tension between clinical reproducibility and explanatory ambiguity

Skeptical literature highlights the paradox that MTP-related phenomena, including localized tenderness, taut bands, and referred pain, are clinically reproducible, yet lack universally accepted biomarkers or diagnostic standards (Quintner et al., 2015; Wilke et al., 2018; Barbero et al., 2019).

Rather than interpreting this variability as evidence against the existence of MTPs, the Protective Model reframes it as consistent with context-dependent physiological responses emerging under strain–repair mismatch. If MTPs represent dynamic expressions of adaptive sentinel response to fluctuating mechanical and physiological stressors, variability in presentation, symptom expression, and detectability would be expected.

### 8.7 Integrative resolution of fragmented explanatory frameworks

Collectively, prevailing models provide valuable but partial insights into MTP physiology, each emphasizing discrete domains such as neuromuscular activity, metabolic stress, sensitization, or biochemical signaling. The Protective Model does not reject these mechanisms but situates them within a unified systems-level framework centered on strain–repair mismatch and adaptive sentinel response.

Within this integrative perspective, neuromuscular contracture, biochemical signaling, sensitization, and metabolic alterations are interpreted not as isolated pathologies, but as coordinated physiological expressions of an adaptive sentinel response arising when cumulative tissue stress exceeds local regenerative capacity. This framework offers a coherent explanation for the formation, persistence, variability, and recurrence of MTPs while remaining consistent with empirical findings across multiple domains of the literature.

## 9 Implications

The introduction of the Protective Model of Myofascial Trigger Points has several implications for how clinicians, researchers, and educators conceptualize musculoskeletal pain, evaluate soft tissue dysfunction, and interpret localized myofascial findings. In contrast to models that primarily emphasize dysfunction, biochemical imbalance, or diagnostic ambiguity, the Protective Model frames MTPs as adaptive sentinel responses, defined as measurable physiological outputs across neuromuscular, biochemical, and neuroimmune systems that emerge under conditions of sustained strain–repair mismatch.

From a clinical perspective, this reframing shifts emphasis away from viewing trigger points solely as pathological entities requiring elimination. If MTPs represent physiological expressions associated with load modulation in mechanically stressed tissue, their presence may warrant investigation into underlying biomechanical, postural, or usage-related factors that perpetuate nociceptive signaling. Interventions targeting the MTP directly, such as needling or manual therapy, may provide short-term symptom relief; however, long-term resolution may be limited if the originating mechanical strain or functional overload remains unaddressed. This interpretation supports a layered therapeutic approach in which MTPs are considered physiological indicators guiding assessment toward primary mechanical or functional contributors.

For diagnostic interpretation, the Protective Model offers a structured explanation for variability in palpation findings, imaging results, and symptom expression. Rather than interpreting inconsistency as evidence of unreliability, this framework suggests that MTP characteristics, including tenderness, stiffness, spontaneous electrical activity, and biochemical signaling, may exist along a spectrum influenced by severity, chronicity, and contextual mechanical load. Emerging elastography research demonstrating measurable changes in tissue stiffness following mechanical intervention supports the view that MTPs are dynamic and structurally responsive phenomena rather than fixed pathological lesions (Szajkowski et al., 2025). Advances in shear wave elastography, localized biochemical assays, and neurophysiological markers may therefore offer stage-sensitive insight into tissue status within regions exhibiting adaptive sentinel responses.

From a research standpoint, the Protective Model provides a systems-level bridge between previously fragmented domains of MTP literature. Rather than positioning MTPs as mutually exclusive with central sensitization, energy crisis, or inflammatory frameworks, the model situates these findings within a coordinated physiological context. Localized tissue stress may be associated with neuromuscular modulation, biochemical signaling, and nociceptive sensitization that co-occur as components of an adaptive sentinel response. This integrative interpretation may help explain why MTPs frequently persist or recur in mechanically overloaded contexts despite localized treatment, a phenomenon not consistently addressed by single-mechanism models.

The model also carries implications for chronic pain research. If persistent MTPs reflect ongoing strain–repair mismatch, they may act as sustained peripheral nociceptive inputs that contribute to the maintenance of central sensitization rather than being solely downstream consequences of central nervous system amplification. Addressing underlying mechanical load and tissue stress in such cases may therefore influence both peripheral and central pain mechanisms, consistent with studies reporting central pain modulation following targeted myofascial interventions.

At a conceptual level, the Protective Model encourages a shift in interpretation from symptom suppression toward physiological system analysis. Within this framework, MTPs are not merely focal pain generators, but measurable indicators of regions where cumulative tissue load exceeds local repair capacity. This perspective does not negate existing pathological interpretations but instead offers an integrative lens through which neuromuscular, biochemical, and sensory findings may be understood as coordinated physiological expressions arising under sustained strain–repair mismatch.

## 10 Limitations

The Protective Model integrates structural, biochemical, neurophysiological, and mechanical findings into a unified framework; however, several limitations must be acknowledged. First, although the model is grounded in established principles of muscle injury, repair physiology, and neuroimmune signaling, the reinterpretation of these mechanisms as coordinated physiological components of an adaptive sentinel response remains theoretical. Direct experimental evidence demonstrating that taut bands or spontaneous electrical activity function to restrict mechanical load or stabilize structurally compromised fibers has not yet been produced. Existing literature describes these phenomena descriptively, but their functional role within a strain–repair mismatch framework has not been empirically validated.

Second, the model assumes that varied forms of muscle damage, including micro-tearing, tensile strain, crush injury, or incisional trauma, engage broadly similar repair cascades capable of producing comparable localized structural and biochemical responses. While this assumption is consistent with general principles of muscle regeneration and inflammatory signaling, the extent to which different injury types yield equivalent contracture-like formations, electrophysiological activity, or biochemical signatures has not been systematically examined.

Third, the proposal that MTPs represent dynamic, spectrum-based physiological expressions rather than discrete latent or active entities challenges existing diagnostic conventions. Current clinical categorization relies heavily on palpation, symptom provocation, and examiner interpretation, which may limit the precision with which model-derived predictions can be tested. Improved assessment methodologies, including high-resolution elastography, advanced intramuscular electromyography, microvascular imaging, and localized biomarker sampling, will likely be necessary to rigorously evaluate several hypotheses generated by the model.

Fourth, chronicity within the model is primarily attributed to persistent mechanical overload or insufficient repair capacity. However, individuals with longstanding MTP-associated pain frequently present with complex psychosocial and systemic physiological factors, including sleep disruption, metabolic dysregulation, hormonal variation, and established central sensitization, all of which may influence local muscle behavior and tissue recovery. While the present model intentionally emphasizes peripheral physiological mechanisms, disentangling the relative contributions of central and peripheral drivers remains a significant challenge for future empirical investigation. Accordingly, the model does not exclude central mechanisms, but instead proposes that sustained peripheral strain–repair mismatch may act as one contributing driver of broader nociceptive amplification.

Fifth, the present work is conceptual and theoretical in scope. It synthesizes existing empirical findings to generate a physiologically coherent and falsifiable framework but does not itself provide experimental validation, diagnostic protocols, or clinical outcome data. Prospective experimental, longitudinal, and interventional studies will be required to test the model’s predictions and evaluate its translational relevance in clinical populations.

Finally, because the model employs an adaptive sentinel response framework, there is a potential risk of teleological misinterpretation. The model does not imply conscious, goal-directed, or intentional behavior of tissues. Rather, it interprets established cellular, neuromuscular, and neuroimmune responses within the context of mechanical stress and repair physiology. Continued refinement and empirical testing will be necessary to ensure that adaptive interpretations remain grounded in measurable physiological processes rather than inferred functional intent.

Despite these limitations, the Protective Model offers a cohesive and testable framework that integrates previously fragmented findings and generates explicit, falsifiable predictions. These limitations therefore delineate key areas for future empirical investigation rather than undermining the conceptual validity of the model.

## 11 Conclusion

Myofascial trigger points remain a clinically significant yet conceptually ambiguous phenomenon. The Protective Model situates MTP-associated findings within established biological stress–repair cascades, conceptualizing them as physiological expressions of an adaptive sentinel response arising from strain–repair mismatch rather than isolated pathological abnormalities. By integrating mechanical, neurophysiological, and neuroimmune observations into a systems-level framework, the model provides a coherent interpretation of previously fragmented empirical findings.

Within this framework, latent and active presentations are reinterpreted as gradations of a single underlying physiological process, with load dynamics and repair capacity acting as primary determinants of persistence, variability, and resolution. Importantly, the model generates explicit, falsifiable predictions related to mechanical modulation, electrophysiological behavior, biochemical signatures, and recurrence patterns, thereby enabling direct empirical evaluation.

The Protective Model does not purport to resolve all uncertainties surrounding MTPs; rather, it offers a biologically grounded and testable framework intended to guide systematic investigation. Future experimental research examining mechanical load modification, temporal progression, and tissue-level physiological measurements will be essential to evaluate the validity, scope, and limitations of the adaptive sentinel response framework in MTP formation and chronicity.

Through iterative empirical testing, this approach may help clarify how coordinated neuromuscular, biochemical, and sensory responses emerge under sustained strain–repair mismatch and contribute to the formation, persistence, and resolution of myofascial trigger points.

## Data Availability

The original contributions presented in the study are included in the article/supplementary material. Further inquiries can be directed to the corresponding author.
